# Humpback whales on maternity leave: when nursing mothers and growing calves can’t afford to rest

**DOI:** 10.1093/conphys/coag078

**Published:** 2026-10-02

**Authors:** John Howieson

**Affiliations:** School of Health and Life Sciences, University of the West of Scotland, Glasgow G72 0LH, UK

Motherhood in mammals is often an energetically demanding feat. Compared with other taxonomic groups, such as amphibians or fish, that reproduce *en masse* to increase the likelihood of at least some offspring surviving mammals tend to produce fewer offspring and invest more heavily in their survival.

The humpback whale (*Megaptera novaeangliae*) takes parental care a step further. Instead of staying put in food-rich polar hunting grounds, humpback whale mothers opt for food-scarce but warm, tropical breeding habitats. While seemingly nonintuitive, this strategy ensures humpback whale mothers conserve energy. The warm waters reduce the energy costs associated with keeping herself and her calf warm and fasting during the breeding season conserves energy that would be spent hunting. However, fasting while nursing makes balancing energy costs a crucial task during the breeding season to ensure survival for both the mother and her calves.

To understand how energy is used by humpback whales on their breeding grounds, Hollers *et al.* (2026) determined the energy costs of mothers and calves off the coast of Hawai’i between 2020 and 25. To do this, biologging tags were attached to 20 mothers and 31 calves (Fig. 1), which recorded swim speed, detected when the whale dived and monitored tailbeats from the individual. These data were complemented by aerial drone footage that was used to help characterise behaviours and record morphometrics such as the size and weight of an individual. The research team were able to calculate the specific energy cost associated with different behaviours for every whale.

**Figure 1:**
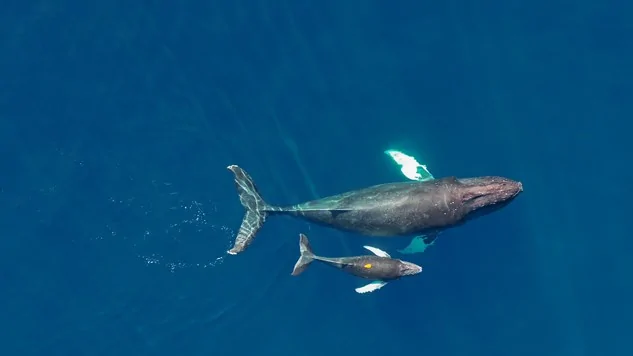
Drone view of a tagged humpback whale calf. Image credit: Lewis Evans.

For both mothers and calves, drifting below the surface (known as resting dives) was found to conserve 35–44% of energy compared to other diving behaviours. Interestingly, older calves spent increasingly more time resting as they aged, likely to offset the energy costs associated with rapid growth. For calves, daily energy expenditure could sit between 92–452 megajoules (a measure of energy) per day depending on the age and size of the individual, while their mothers would expend between 817and 2013 megajoules per day, all while fasting and nursing their calves!

In contrast, simulated models show that disturbance of these breeding grounds could increase the daily energy expenditure up to 8.5%, which would require the mother to eat an additional 43.6 kg of krill in the feeding season to offset a day of disturbance. Ensuring this trade-off is balanced becomes even more difficult for these mother and calf pairs when key habitats are facing environmental changes and anthropogenic disturbances. Where anthropogenic disturbances (such as passing fishing or whale watching vessels) can disrupt nursing or incite avoidance dives, these stressors can add further pressure onto mothers which are already running on empty.

As climate change associated heatwaves occur in feeding grounds, the prey needed to sustain female humpback whales during the breeding season is declining. Combined with disturbances from fishing or whale-watching vessels, humpback whale mothers may increasingly find themselves drained of essential energy reserves. Hollers *et al.* (2026) highlight the need to protect these key breeding grounds to ensure that energy reserves that help sustain mothers and nurture calves are maintained during this critical period in the humpback whale lifecycle.

## Notes

**Editor**: Ashleigh Donaldson
